# Training Effects of the FIFA 11+ Kids on Physical Performance in Youth Football Players: A Randomized Control Trial

**DOI:** 10.3389/fped.2018.00040

**Published:** 2018-03-05

**Authors:** Carlos Pomares-Noguera, Francisco Ayala, Francisco Javier Robles-Palazón, Juan F. Alomoto-Burneo, Alejandro López-Valenciano, José L. L. Elvira, Sergio Hernández-Sánchez, Mark De Ste Croix

**Affiliations:** ^1^Sports Research Centre, Miguel Hernández University of Elche, Alicante, Spain; ^2^Centre for Translational Research in Physiotherapy, Department of Pathology and Surgery, Physiotherapy Area, Miguel Hernández University of Elche, Alicante, Spain; ^3^School of Sport and Exercise, University of Gloucestershire, Gloucester, United Kingdom

**Keywords:** youth athletes, agility, injury prevention, jumping, warm-up

## Abstract

**Objective:**

To analyze the training effects of the FIFA 11+ kids on several parameters of physical performance in male youth football players.

**Materials and methods:**

Twenty-three youth players were randomized within each team into two groups (control vs. intervention). The intervention group performed the FIFA 11+ kids programme 2 times a week for 4 weeks; the control groups completed their normal warm-up routines. Thirteen physical performance measures {range of motion (hip, knee, and ankle joints), dynamic postural control (measured throughout the Y balance test), 20 m sprint time, slalom dribble with a ball, agility, vertical jumping height [counter movement jump (CMJ) and drop jump (DJ)], horizontal jump distance, accuracy when volleying a ball [measured throughout the Wall Volley test]} were assessed. All physical performance parameters were compared *via* magnitude-based inference analysis.

**Results:**

Significant between-group differences in favor of the FIFA 11+ players were found for dynamic postural control {anterior [mean and 90% confidence intervals (CI) = 1 cm, from −1.6 to 3.5 cm] and posteromedial (mean and 90% CI = 5.1 cm, from −1.8 to 12 cm) and posterolateral (mean and 90% CI = 4.8 cm, from 0.6 to 9.0 cm) distances}, agility run (mean and 90% CI = 0.5 s, from −0.9 to 0 s), vertical jump height [CMJ (mean and 90% CI = 3.1 cm, from 0.2 to 6.1 cm) and DJ (mean and 90% CI = 1.7 cm, from −0.5 to 3.9 cm)], and horizontal jump distance (mean and 90% CI = 2.5 cm, from −8 to 15 cm). The control groups showed better performance in 20 m sprint time (mean and 90% CI = −0.05 s, from −0.11 to 0.07) and wall volley tests (mean and 90% CI = 0.2, from −0.2 to 0.6) compared to the intervention group.

**Conclusion:**

The main findings of this study suggest that just 4 weeks of implementation of the FIFA 11+ kids produces improved physical performance compared with traditional warm-up routines in youth soccer players.

## Introduction

Football (soccer) is a physically demanding sport that entails sudden acceleration and deceleration, rapid changes of directions, jumping and landing tasks, as well as many situations in which players are involved in tackling to keep possession of or to win the ball (1, 2). These high-intensity situations result in a notable increase in injury risk and are especially relevant in children where individual growth and maturation may predispose youth players to a higher risk (3–5). Epidemiology studies have reported that the frequency and severity of injuries among youth football players is striking in comparison to other sports (6–8). In particular, a recent epidemiological study of young children (7–12 years of age) reported injury incidence rates of 0.61 per 1,000 h of exposure and an increase in the incidence rate with age (9). While some studies have explored position-related differences in injury incidence (10), it should be noted that there may be differing movement demands based on football position; however, no studies have directly explored this within youth football.

Therefore, it would appear important to implement effective injury prevention programmes early to counter potential injury-related risks. Several injury prevention programmes have been designed with the aim of preventing and reducing the number and severity of football-related injuries in adolescent players, such as the FIFA 11+, knee injury prevention program and preventing injury and enhancing performance program (11–14). All of these programmes include running exercises and specific dynamic movements focusing on enhancing physical competence and performance, as it has been suggested as a primary injury risk factors in youth athletes (15) and are based on the injury profile and biological status (i.e., maturation phase) of the target population (>14 years of age). The effectiveness in reducing non-contact overall and overuse lower extremity injury rates of these abovementioned injury prevention programmes have been documented in male and female youth (aged 13–19 years) players (16).

However, these injury prevention programmes may not be suitable for younger players (<13 years of age) since maturation seems to affect the incidence and characteristics of injury. Specifically, injury incidence in youth populations has been shown to be higher in adolescents than pre/early pubertal players (17). Thus, injury incidence has recently been aligned to peak height velocity (adolescence), when rapid disproportional growth is evident (18). Furthermore, younger football players seem to have more fractures and bone stress, fewer strains and sprains, and more injuries of the upper body than adolescent players (9). These considerations have led some authors to develop a warm-up programme designed to prevent injuries and reduce the number and severity of football-related injuries in children who take age-specific injury characteristics and physical maturity into account (19). The warm-up programme “FIFA 11+ Kids” is intended to prevent and reduce the number and severity of football-related injuries by enhancing children’s fundamental and sport-specific motor skills through a range of evidence-based exercises. In particular, the FIFA 11+ kids focuses on: (a) spatial orientation, anticipation, and attention, particularly while dual-tasking (to avoid unintended contact with other players or objects); (b) body stability and movement coordination (more general than specific neuromuscular or proprioceptive training); and (c) learning appropriate fall techniques (to minimize the consequences of unavoidable falls). Accordingly, this warm-up programme may contribute to achieving the aims of the earlier stages [(1) Active Start, (2) FUNdamentals, and (3) Learn to Train] of the long-term athletic development conceptual model for late specialization sports (20, 21).

Only one large-scale cluster-randomized controlled trial has evaluated the effectiveness of the FIFA 11+ kids showing a 38% reduction in injuries, with severe injuries reduced by more than 50%, compared to a control group (data available at https://www.fifamedicinediploma.com/lessons/prevention-fifa11-kids/).

However, the training effects elicited on movement skills and physical performance on the potential mechanisms behind the reported reduction in injury incidence remain to be elucidated. To the best of our knowledge, only some authors have examined the pre-exercise effects of the FIFA 11+ kids on various physical performance variables in children football players, showing possibly beneficial effects in static and dynamic balance, jumping performance, and slalom dribbling (19).

Therefore, the main purpose of this study was to analyze the training effects of the FIFA 11+ kids on several parameters of physical performance in young football players. We hypothesized that this new program would show beneficial and superior effects on physical performance (particularly in balance and agility measures) in comparison to the traditional practices as they include specific and novel exercises designed to improve physical competency.

## Materials and Methods

### Research Design and Participants

A total of 26 male youth football players (age: 11.8 ± 0.3 years; stature: 144.7 ± 5.1 cm; body mass: 39.4 ± 5.5 kg) took part in the current study. Participants were recruited from two different football teams that were engaged in the Official Amateur Championships of the Spanish Football Federation (under 12 years regional league). All participants were classified as pre-peak height velocity (PHV) (−3 years to −1 years from PHV) and met three inclusion criteria: (1) had no history of impairments to the knee, thigh, hip, or lower back in the 6 months prior to the study; (2) all participants were free of self-reported delayed onset muscle soreness at any testing session (self-reported); and (3) participated in two training sessions per week (1.5–2 h per session). In addition, two exclusion criteria were also established: (1) missed two consecutive or three non-consecutive training sessions and (2) missed one testing session (22).

All experimental procedures and potential risks were explained fully both verbally and in writing to the participants, and written informed consent was obtained from players, their parent/guardian, and coaches. The Institutional Research Ethics committee of the Miguel Hernandez University of Elche (DPS.FAR.01.14), conforming to the recommendations of the Declaration of Helsinki, approved the study protocol prior data collection.

Twenty-three young football players from two different football teams completed this study. Three players who belonged to one team were excluded from the study because they missed more than three non-consecutive training sessions. The participants’ characteristics can be observed in Table 1.

**Table 1:** Demographic variables for the children football players^a^.

Age (years)	11.8 ± 0.3
Stature (cm)	144.7 ± 5.1
Body mass (kg)	39.4 ± 5.5
Body mass index (kg/m^2^)	18.7 ± 2.1
Maturation offset (years from pre-peak height velocity)	−2.4 ± 0.4

*^a^All values are mean ± SD*.

A parallel, two-group, pre-post, randomized controlled trial with double baseline (two pre-test sessions) was used to address the purposes of this study.

The study was conducted between February and April 2015. In Spain, the under 12 local league has two rest periods [winter (2–3 weeks for Christmas holidays) and spring (2 weeks for Easter holidays) breaks] so the season is divided into three main terms/macrocycles. The three terms have approximately the same number of weeks (8–10 weeks) and matches (8–10 matches; one every weekend). The time frame of the study was selected so that the study started after the winter break and could be completed before the Easter break. The second term of the season was chosen rather than the first term in order to be sure that the players selected to each team was definitive and stable within the testing period. Further, the study was not carried out in the third term of the season with the aim of reducing the dropout rate of players’ that could be expected due to the primary school final exams.

The independent variables were the two different warm-up programmes [control (traditional or regular warm-up) and intervention (FIFA 11+ kids)]. The dependent variables included 13 physical performance measures {range of motion (hip, knee, and ankle joints), dynamic postural control (measured throughout the Y balance test), 20 m sprint time, vertical jump height [counter movement jump (CMJ) and drop jump (DJ)], horizontal jump distance, accuracy when volleying a ball (measured throughout the wall volley test), slalom dribble and agility}.

Prior to the intervention phase, the participants’ baseline value for each dependent variable was determined using two identical testing sessions separated by a week rest-interval. Each testing session was carried out 48–72 h after finishing the previous competitive match (i.e., Tuesday or Wednesday) so that the players had enough time for recovery. In addition, players did not carry out any training session throughout this rest-interval. Tests were conducted within the time frame of a regular training session at the same time of the day (in the late afternoon). All the tests were carried out on an outdoor training pitch (3G artificial surface). The total testing procedure lasted approximately 2 h for one team. After the two pre-test sessions were completed, participants were randomized within each team into two groups [team 1: control (*n* = 6) vs. FIFA 11+ kids (*n* = 6); team 2: control (*n* = 4) vs. FIFA 11+ kids (*n* = 7)] using a computer-based software programme. One of the researchers without any contact or knowledge of the players completed the allocation and randomization. Therefore, no allocation concealment mechanisms were necessary.

For the following 4 weeks (intervention phase), the participants completed only one of the two intervention programmes 2 days a week as part of their weekly training sessions. Prior to competitive matches, all players performed their normal warm-up routines (this was imposed by the coaches of both teams).

The training period of 4 weeks was selected (a) to match the typical duration of each of the two mesocycle that make up the three macrocycles of the regular season and (b) to ensure that both the testing and intervention phases of the study were completed during the same period of the season in each team.

A Master in Sports Science student was assigned to both teams for administrating the FIFA 11+ kids and for checking that the coaches delivered their normal warm-ups in the control group. All players in the intervention groups attended a workshop designed to demonstrate how to perform the exercises correctly. In order to prevent contamination of the control groups, the training pitch was divided into two equal parts, so that the players who belonged to the control group performed their regular warm-up in one part while the players who belonged to the intervention group performed their new warm-up in another part of the pitch.

Two days after the intervention phase, the post-intervention assessments were carried out following the same procedure completed during the baseline-testing phase. Due to organizational reasons, the same two testers (one tester conducted the tests while the other tester recorded the data) who conducted the baseline and post-intervention assessments were not blinded to group assignment.

### Testing Procedure

**Figure 1:**
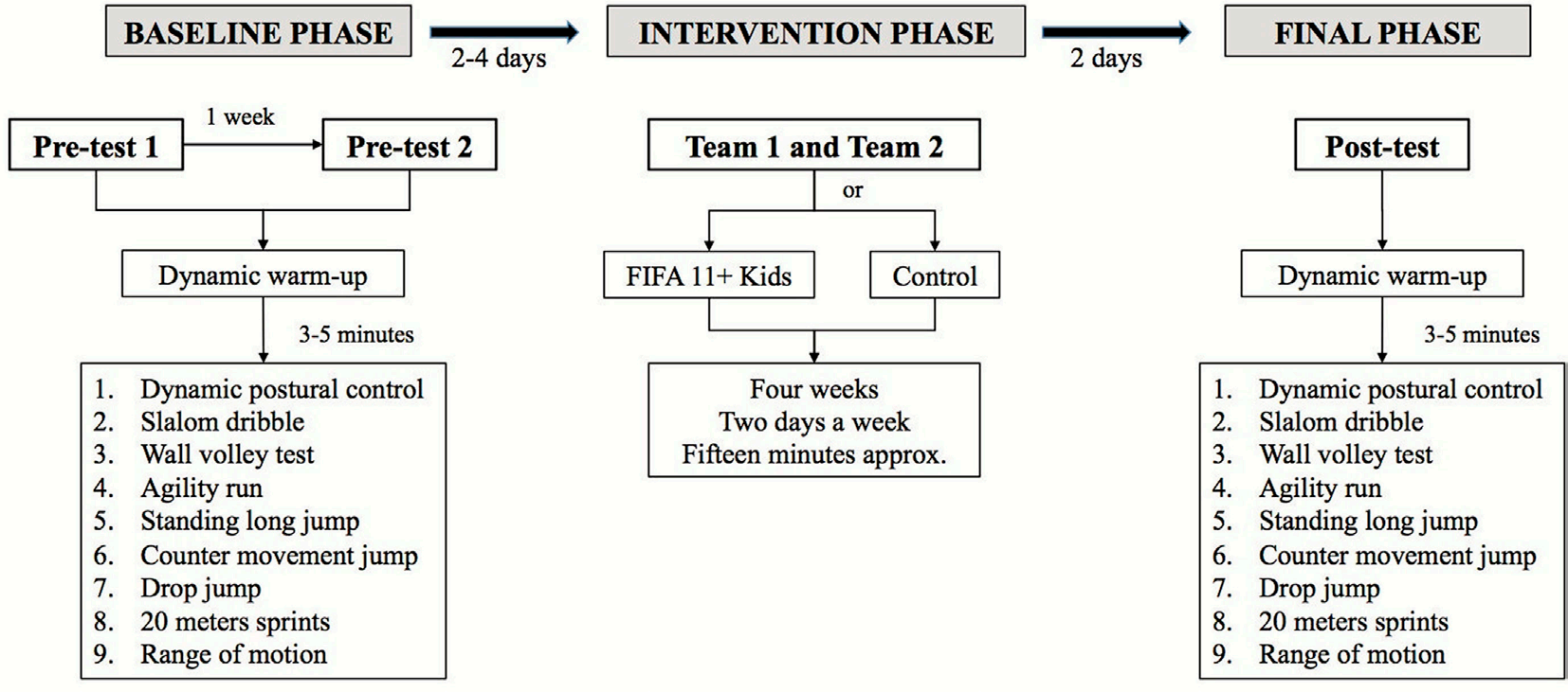
Schematic representation of the study design.

During each testing session, participants began by completing a standardized warm-up routine consisting of 4–5 min of self-paced low- to moderate-intensity running including forward/backward movements, sidestepping, and general mobilization (i.e., arm circles, leg kicks). After this, participants performed 6–8 min of dynamic stretching (i.e., straight leg march, forward lunge with opposite arm reach, forward lunge with an elbow instep, lateral lunge, trunk rotations, multidirectional skippings) performing three sets, from low to high intensity, with a 15 s rest period between each set. The assessments of the dependent variables were carried out 3–5 min after the standardized warm-up. The order of the tests was consistent through the experimental sessions and is displayed in Figure 1.

#### Dynamic Postural Control

Dynamic postural control was evaluated using the Y balance test. Players were allowed a maximum of five trials to obtain three successful trials for each reach direction (anterior, posteromedial, and posterolateral). Trials were discarded if the player failed to maintain unilateral stance on the platform, failed to maintain reach foot contact with the reach indicator on the target area while the reach indicator is in motion, used the reach indicator for stance support, or failed to return the reach foot to the starting position under control (23). Specifically, testing order was completed as dominant anterior, non-dominant anterior, dominant posteromedial, non-dominant posteromedial, dominant posterolateral, and non-dominant posterolateral. The best of the three reaches was normalized by dividing by leg length to standardize the maximum reach distance ((excursion distance/leg length) × 100 = % maximum reach distance) (24). Leg length was defined as the length measured in centimeters from the anterior superior iliac spine to the most distal portion of the medial tibial malleolus. To obtain a global measure of the balance performance, data from each direction was averaged to determine a composite score (24).

#### 20-m Sprint

Time during a 20-m sprint in a straight line was measured by means of single beam photocell gates placed 0.3 m above the ground level (Time It; Eleiko Sport, Halmstad, Sweden). Each sprint was initiated from an individually chosen standing position, 30 cm behind the photocell gate, which started a digital timer. Each player performed three maximal 20 m sprints interspersed with 3 min of passive recovery, and the fastest time achieved was retained (25).

#### Agility

The Illinois agility test is commonly used in measuring agility in football (19, 26, 27). The length of the zone is 10 m, while the width (distance between the start and finish points) is 5 m. Four cones were placed in the center of the testing area at a distance of 3.3 m from one another. Four cones were used to mark the start, finish, and two turning points. The participants started the test lying face down, with their hands at shoulder level. The trial started on the “go” command, and the participants began to run as fast as possible. The trial was completed when the players crossed the finish line without having knocked any cones over. Time was measured using a photocell system (Time It; Eleiko Sport, Halmstad, Sweden). Each player performed three trials with the best score (time) used for analysis.

#### Slalom Dribble

The slalom dribble course was 20 m in length. Participants ran with the ball in a zig-zag fashion around five cones placed in a straight line 4.5 m away from one another. The run time was measured using two photoelectric timing gates. Participants started 0.3 m in front of the starting line and performed four repetitions with the best score (time) used for analysis.

#### Wall Volley Test

The wall volley test required players to pass the ball through the air against a wall, control the rebound, and make as many direct air-borne passes against the wall as possible, within a time limit of 30 s. The outcome was the absolute number of correct rebounds (28). The player was placed in a field which was 2 m wide and 0.5 m away from the wall. Only rebounds accomplished while standing in the sector were counted. After two familiarization tests, participants completed two repetitions and the best score was used for analysis.

#### Standing Long Jump

The participant stood behind the starting line and was instructed to push off vigorously and jump as far as possible. The participant had to land with the feet together and to stay upright. Arm swing was not permitted. Jump distance was measured from the take off line to the back of the heel. After two familiarization tests, participants performed three repetitions. The best score of the three repetitions was selected for the subsequent analysis.

#### Counter Movement Jump

A CMJ without arm swing was performed on a contact platform (Ergojump1, Finland) (29). During the CMJ, the participants first stood upright, then squatted to a self-selected depth of approximately 90° knee flexion, and jumped immediately as high as possible. Players were asked to keep their hands on their hips to prevent the influence of arm movements on vertical jump performance. In addition, players were allowed to perform a countermovement with the lower limbs before jumping. Each player performed five maximal CMJs interspersed with 45 s of passive recovery, and the best jump height was recorded.

#### Drop Jump

A vertical DJ without arm swing was performed on a contact platform (Ergojump^®^, Finland) (30). Participants stood with feet shoulder-width apart on a 28-cm-high step, 30 cm from the contact platform. They were instructed to lean forward and drop from the step as vertically as possible, in an attempt to standardize landing height. Participants were required to land with one foot on each of the force plates, then immediately perform a maximal vertical jump, finally landing back on the contact platform. Participants were asked to keep their hands on their hips to prevent the influence of arm movements on vertical jump performance. Each participant performed at least five maximal jumps starting from a standing position, with at least 1 min of recovery between jumps. Participants were asked to jump as high as possible with the shortest contact time with the ground. The best jump height was used for statistical analysis.

#### Hip, Knee, and Ankle Range of Motions

Passive hip flexion [passive straight leg raise test], knee flexion [Modified Thomas test], and ankle dorsiflexion [weight-bearing lunge with knee extended test] range of motion of the dominant and non-dominant extremities were assessed (31). Participants were barefoot and instructed to perform in a randomized order and two maximal trials of each range of motion test for each extremity. The best score for each test was used in the subsequent analyses. The same researchers performed the ROM testing at all testing sessions.

### Interventions

#### Control Group

Coaches were asked to administer their normal warm-up routines trying to match the duration of the FIFA 11+ kids (15–20 min). The traditional warm-up differed slightly between teams but included a combination of running, stretching, technical exercises with the ball and games.

### FIFA 11+ Kids

The FIFA 11+ kids consisted of seven different exercises: a running game, two jumping exercises, a balance/coordination task, two exercises targeting body stability, and an exercise to improve falling technique (19). The program has a modular structure and consists of three skill levels with progressive load. The players completed the FIFA 11+ kids two times a week for 4 weeks substituting their normal warm-up routine. All players were able to perform the level II of difficulty for each exercise in part 2 properly as confirmed during the habituation workshop. However, no player was able to perform the level III of difficulty for each exercise, and therefore, the level II of difficulty was used.

### Statistical Analysis

The distribution of raw data sets was checked using the Kolmogorov–Smirnov test and demonstrated that all data had a normal distribution (*p* > 0.05).

Dependent sample *t*-tests were carried out to assess differences between limbs (dominant vs. non-dominant) in dynamic postural control and range of motion pre-test 1 and pre-test 2. In cases where no significant differences were found, the mean value of both limbs was used for the subsequent analyses. Dependent *t*-tests were also carried out to assess baseline inter-session differences (pre-test 1 vs. pre-test 2) for each dependent variable. If no significant differences were found, the mean value of both testing sessions for each variable was used to assess the effects of the intervention programs. Contrarily, if significant differences were found, the highest value of both testing sessions for each variable was used for the magnitude-based inference analysis of the interventions. Independent sample *t*-tests were run to evaluate baseline differences between the groups (intervention vs. control) for each dependent variable (mean of the two baseline measures).

Magnitude-based inference analysis of the warm-up programmes (intervention and control) were estimated using a spreadsheet *via* Student’s *t*-test with unequal variances computed for change scores between paired sessions (intervention vs. control) at each testing time [pre-test (baseline), post-test] for each variable (32). Alpha was set at *p* < 0.05. Each participant’s change score between pre- and post-tests was expressed as a percentage of baseline score *via* analysis of log-transformed values, to reduce bias arising from non-uniformity of error.

This approach of data analysis uses confidence intervals (CI) to calculate the probability that a difference is of practical relevance or trivial when a value for the smallest worthwhile change is entered. A difference score of at least 0.2 of the between-participant SD (representing a small effect) was considered to be practically worthwhile (33). The qualitative descriptors were used to interpret the probabilities that the true affects are harmful, trivial, or beneficial: <1%, almost certainly not; 1–4%, very unlikely; 5–24%, unlikely or probably not; 25–74%, possibly or may be; 75–94%, likely or probably; 95–99%, very likely; >99%, almost certainly (34). This spreadsheet also provides estimates of the effect of an intervention adjusted to any chosen value of the covariate, thereby reducing the possibility for confounding of the effect when a characteristic is unequal in the experimental and control groups: thus, the baseline pre-test value (mean of the two pre-test measures) of each dependent variable was included to avoid the phenomenon of regression to the mean and thereby obtaining a better estimation of the effects of the warm-up programmes in comparison with their paired control groups.

## Results

*t*-Tests demonstrated no significant differences (*p* values from 0.06 to 0.97) in Y balance test (anterior, posteromedial, and posterolateral directions and composite score) and ROM (hip flexion, knee flexion, and ankle dorsiflexion) outcomes between the dominant and non-dominant limbs of the players at either pre-test 1 or pre-test 2. Consequently, the average score of both limbs for each unilateral variable was used for the subsequent statistical analysis. No significant differences (*p* values from 0.08 to 0.97) were found between the scores obtained in both pre-test sessions in each variable (with the exception of the Illinois test [mean pre-test 1 vs. pre-test 2 difference = 0.24 ± 0.42 s; *p* = 0.01]) so the average score was used as criterion of reference (baseline score) (Table 2). In addition, there were no paired inter-group differences at baseline for any dependent variable (*p* values ranging from 0.24 to 0.89). The pre (baseline) and post-intervention results of each group are reported for descriptive purposes in Table 3 (FIFA 11+ kids and control).

**Table 2:** Descriptive pre-tests statistics (mean ± SD) and differences between pre-test 2 and pre-tests 1 (mean ± 95% confidence intervals).

Variable	Pre-test 1		Pre-test 2		Differences			*p* level
**Y balance test^a^**								
■ Anterior distance	75.1	±12.1	75.8	±10.7	0.7	(−2.5 to 3.9)		0.63
■ Posteromedial distance	107.7	±14.0	108.9	±12.9	1.2	(−2.1 to 4.6)		0.44
■ Posterolateral distance	110.5	±17.9	111.5	±15.6	1.0	(−2.3 to 4.4)		0.52
■ Composite	97.8	±13.3	98.8	±12.2	1.0	(−1.2 to 3.2)		0.65
20 m sprint time (s)^b^	3.83	±0.18	3.81	±0.18	−0.02	(−0.09 to 0.04)		0.51
Agility run (s)^b^	19.97	±0.77	19.72	±0.76	−0.24	(−0.43 to 0.06)		0.01
Slalom dribble (s)^b^	6.12	±0.34	6.14	±0.39	0.02	(−0.16 to 0.20)		0.82
Wall volley (*n*)	3.0	±0.9	2.7	±0.4	−0.3	(−0.6 to 0.1)		0.08
Standing long jump (cm)	142.6	±10.8	142.6	±14.9	0.0	(−3,6 to 3.7)		0.97
Counter movement jump (cm)	23.2	±3.4	22.9	±2.5	−0.3	(−0.7 to 0.1)		0.59
Drop jump (cm)	19.7	±2.9	20.5	±3.3	0.8	(−0.2 to 1.9)		0.13
**Range of motion (°)**								
■ Hip flexion	71.6	±5.2	71.7	±5.3	0.1	(−0.1 to 0.4)		0.27
■ Knee flexion	129.8	±5.9	129.9	±6.1	0.1	(−0.2 to 0.5)		0.53
■ Ankle dorsiflexion	31.3	±3.9	31.3	±3.8	0	(−0.4 to 0.4)		1.0

*°, degrees; s, seconds; cm, centimeter*.

*^a^Normalized to limb length expressed as a percentage*.

*^b^Smaller values represent better results*.

**Table 3:** Baseline (pre-test) and post-intervention (FIFA 11+ and control) results (mean ± SD) for physical performance outcomes.

Physical performance measure	FIFA 11+ kids						Control					
	Baseline		Post-test		Difference		Baseline		Post-test		Difference	
	Mean	SD	Mean	SD	Mean	95% CI	Mean	SD	Mean	SD	Mean	95% CI
**Y Balance test^a^**												
■ Anterior distance	75.8	± 14.2	77.2	± 12.8	1.4	(−0.2 to 3.2)	75.1	± 14.2	75.6	± 5.6	0.5	(−1.5 to 2.7)
■ Posteromedial distance	109.5	± 14.4	112.9	± 14.9	3.5	(−1.1 to 8.4)	106.9	± 11.3	106.3	± 9.9	−0.6	(−6.9 to 4.0)
■ Posterolateral distance	113.1	± 19.8	116.2	± 17.6	3.1	(−0.1 to 7.1)	108.3	± 10.7	107.4	± 9.9	−0.9	(−3.7 to 1.2)
■ Composite	99.5	± 15.5	102.1	± 14.3	2.7	(−0.1 to 5.7)	96.7	± 7.5	96.4	± 7.4	−0.3	(−3.3 to 2.1)
20 m sprint time (s)^b^	3.79	± 0.21	3.78	± 0.25	−0.01	(−0.05 to 0.03)	3.87	± 0.06	3.83	± 0.11	−0.04	(−0.1 to −0.01)
Agility run (s)^b^	19.95	± 0.77	19.54	± 0.81	−0.41	(−0.62 to −0.17)*	19.72	± 0.71	19.79	± 0.92	0.07	(−0.37 to 0.47)
Slalom dribble (s)^b^	6.13	± 0.32	5.42	± 0.33	−0.71	(−0.86 to −0.56)*	6.13	± 0.29	5.43*	± 0.38	−0.07	(−0.9 to −0.5)
Wall volley (*n*)	2.9	± 0.6	3.1	± 0.5	0.2	(−0.09 to 0.41)	2.8	± 0.6	3.2	± 0.7	0.4	(0.04 to 0.75)
Standing long jump (cm)	143.0	± 15.5	146.1	± 19.6	3.1	(−5.5 to 10.7)	142.1	± 6.8	141.7	± 10.2	−0.3	(−10.9 to 19.1)
Counter movement jump (cm)	22.7	± 2.9	24.5	± 5.3	1.8	(0.6 to 4.1)	23.5	± 2.7	21.8	± 3.2	−1.7	(−3.1 to 1.5)
Drop jump (cm)	19.8	± 3.2	21.6	± 4.2	1.8	(0.3 to 3.2)	20.5	± 2.4	20.4	± 3.2	−0.1	(−1.7 to 1.6)
**Range of motion (°)**												
■ Hip flexion	72.1	± 6.0	73.8	± 6.6	1.7	(−1.1 to 4.9)	71.1	± 4.2	72.1	± 4.0	1.0	(−0.3 to 2.2)
■ Knee flexion	129.7	± 6.8	131.1	± 6.8	1.4	(−0.7 to 3.4)	130.1	± 4.9	129.1	± 4.4	−1.0	(−3.5 to 1.9)
■ Ankle dorsiflexion	30.9	± 3.9	32.5	± 4.5	2.6	(−0.4 to 3.3)	31.9	± 3.9	33.6	± 3.9	1.7	(−0.5 to 5.1)

*The differences between pre- and post-test average values are also reported (mean and 95% confidence intervals [CI])*.

*^a^Normalized to limb length expressed as a percentage*.

*^b^Smaller values represent better results*.

*p < 0.05.

*s, seconds; cm, centimeter; °, degrees*.

**Figure 2:**
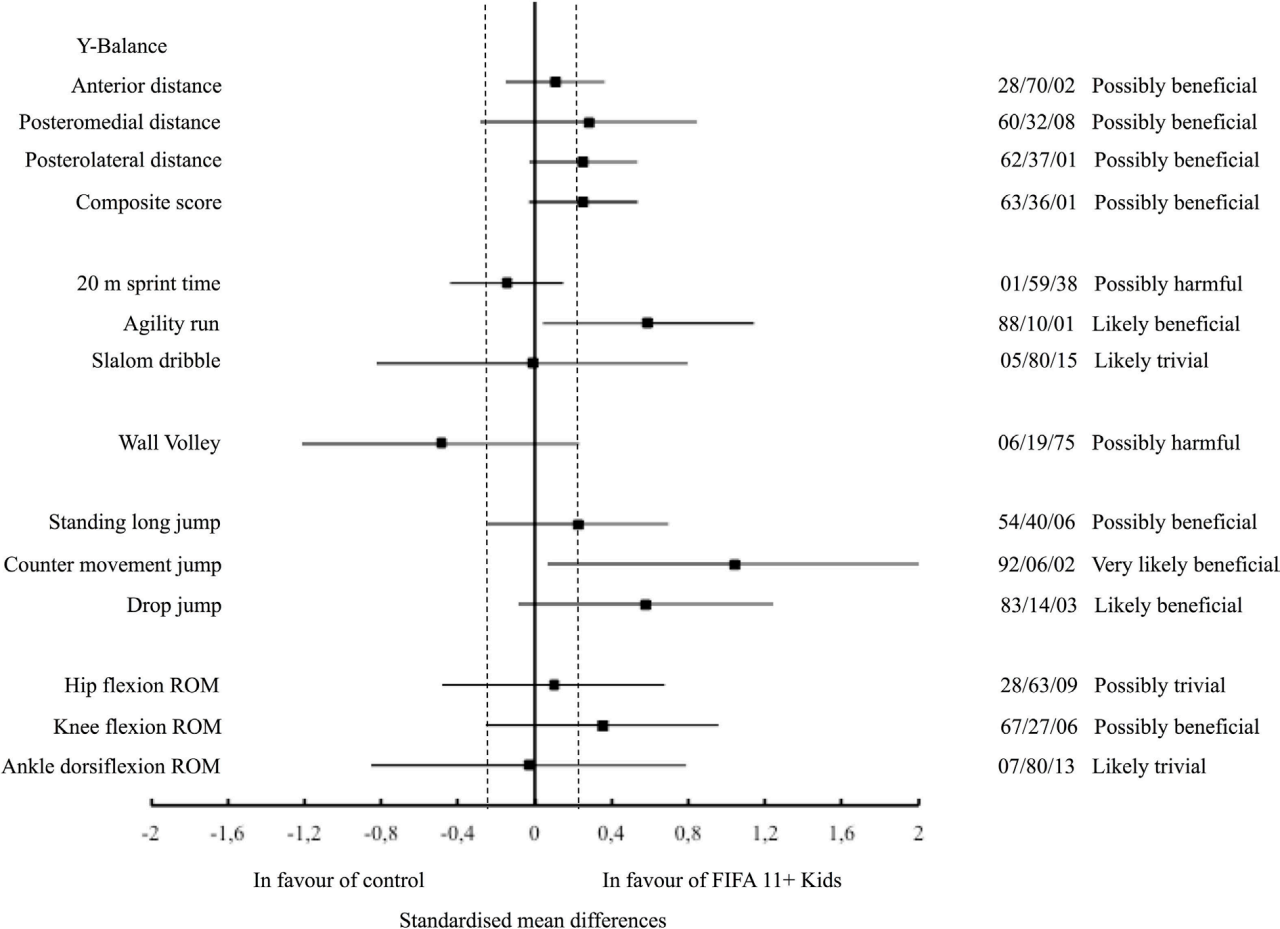
Standardized mean differences (90% confidence interval) between the FIFA 11+. Kids and control group in the physical performance parameters analyzed.

The inter-groups differences after the intervention phase (4 weeks) with the corresponding 90% CI for the physical performance measures are displayed in Figure 2. Very likely and likely beneficial effects favoring the intervention group were observed in CMJ height and DJ height, respectively. Possibly beneficial effects in favor of the intervention group were observed in Y balance test (anterior, posteromedial, and posterolateral distances and composite score), agility run, standing long jump, and knee flexion ROM measures. Likely trivial effects were found for Slalom dribble and for hip flexion and ankle dorsiflexion ROM measures. The control group showed better performance in 20 m sprint time and wall volley tests compared to the intervention group.

## Discussion

The findings of the current study indicate that the training stimuli provided by the implementation of the FIFA 11+ kids two times per week for 4 weeks (8 sessions) appears to be sufficient to elicit small to moderate improvements in some [dynamic postural control, agility run, and jumping (standing long jump, CMJ, and DJ) measures] but not all [20 m sprint time, slalom dribble, wall volley, and ROMs (with the exception of the knee flexion ROM) measures] of the physical performance parameters analyzed.

Similar improvements in dynamic postural control (Y balance anterior distance), agility run and jumping measures have been reported after the implementation of the FIFA 11+ kids twice a week for 10 weeks in a large cohort of young football players (19). However, and in contract to our results, same authors found improvements in slalom dribble and wall volley measures. Perhaps, a possible explanation for these conflicting results might be attributed to the different duration of the intervention phases (10 vs. 4 weeks). Therefore, given the intensity and volume of the FIFA 11+ kids programme used in the current study, 4 weeks (8 sessions) might not be enough to elicit training responses in sprinting times and football-specific coordinative tasks measures (slalom dribble and wall volley).

Force platforms have been used as a criterion measure in scientific setting to assess postural control by interpretation of parameters derived from the centre of pressure (COP) such as velocity and area of COP displacement (35). However, because of its high cost and the need for sophisticated instruments, qualified technicians, and time constraints, the use of this method is limited in sports settings. Consequently, the field-based Y balance test has been recommended as an alternative to force platforms for estimating dynamic postural control in sports setting because: (a) it offers sufficient challenge for dynamic postural control as the subject must maintain balance on a single limb, whilst the other limb carries out a series of reaching tasks (operationally valid); (b) its procedure is simple to administer; (c) instructions are easy to follow; (d) scores are easy to explain; (e) the movements require minimal skills training; and (f) large numbers can be tested in a short period of time (24). Furthermore, the Y balance test has been shown to be sensitive enough: (a) to detect dynamic postural control deficits in patients with chronic ankle instability (36), patellofemoral pain syndrome (37) and anterior cruciate ligament deficiency (38); (b) to identify athletes at high risk of non-contact lower extremity injury (39, 40); and (c) to monitor the rehabilitation and return to play processes (41). Furthermore, the test has also been shown to have high intra and inter-tester reliability (42).

Studies looking at the influence of the FIFA 11+ in adult males have reported improvements in dynamic postural control, agility run, jump performance, and balance (11). The very likely positive effects of jump performance in the current study show probable improvements in power in this group of young children. The possibly beneficial effects of the FIFA 11+ kids on balance and agility found in the current study are important as balance and coordination develop throughout maturation (43). To our knowledge this is the first study to show improvements in balance and agility in prepubertal children following the introduction of a short, focused warm-up programme for only 4 weeks. These findings highlight the benefits of such programmes to development movement competency in young children, which is essential for athletic performance and which forms the foundation of the youth physical development model (44) and also for injury prevention.

As this is the first study (to the authors’ knowledge) to explore the effects of the FIFA 11+ kids on ROM measures, we are not able to make comparisons. The absence of improvements in the joint ROM measures for the ankle and hip, after performing the FIFA 11+ kids was expected because this program does not include any specific group of exercises *a priori* designed to enhance their ROM. However, the possibly beneficial increase in knee ROM is encouraging, given that restricted knee flexion ROM on landing is a risk factor for high ligament loading.

The findings of the current study speculate that the reported improvements in physical performance elicited by the FIFA 11+ kids programme may contribute to a reduction of injury risk in the long-term application. Thus, the mechanisms behind the promising reduction in injury risk by adhering to the FIFA 11+ kids in prepubertal football players might be particularly associated with enhancements in tendon stiffness, balance coordination and physical competency.

### Perspectives and Limitations

An important limitation was the small sample size used in each group (interventions or controls). However, despite the sample size that was enrolled in each group significant main effects were still found. In order to minimize the error associated with the players who belonged to the control group copying and performing any new exercise included in the intervention groups during their regular warm-up, the players of the intervention groups performed their new warm-up in a separate part of the pitch. In addition, a trained rehabilitation specialist was assigned to each team for administrating the interventions and for checking that the control groups did not perform exercise that were not part of their normal warm-up. However, we cannot totally exclude the possibility that the players of the control groups might have performed exercise included in the interventions outside of their regular training sessions. Likewise, and in order to avoid the possible expectation bias of the intervention and control groups, participants did not received information regarding which warm-up programme (FIFA11+ kids or tradition warm-up) was expected to achieve better results in the dependent variables. As we mentioned before, we can neither excluded the possibility that the participants might have sought information about the potential effects of the FIFA kids program on physical performance reported in previous studies. However, neither trainers nor any member of the research staff reported any suspicion about this issue and hence, we consider that this potential source of bias was negligible.

Future studies that investigate the effects of longer interventions than that conducted in the current study (>4 weeks) on several physical performance variables using randomized control trial designs are needed to understand better potential mechanisms behind the reported reduction in injury incidence reported by the FIFA 11+ kids.

## Conclusion

Given the improvements in jump performance, balance, and agility, our study would advocate the introduction of these essential movement competency skills in prepubertal children. It would appear that a structured warm-up routine, such as the FIFA 11+ kids, focusing on balance, jumping/landing, coordination, and stability should be advocated at an early age. These movements should be focused and delivered early in any long-term athlete development model to enhance movement literacy in young athletes (45).

## Author Contributions

CP-N and FA: substantial contribution to the design of the work. CP-N, FR-P, JA-B, and AL-V: acquisition of the data. JE, SH-S, and MD: to revise the work critically for important intellectual content. FA and MD: to provide approval for publication.

## Conflict of Interest Statement

The authors declare that the research was conducted in the absence of any commercial or financial relationships that could be construed as a potential conflict of interest.
